# Effect of Mutations in the C-Terminal 22–24 Domains of Filamin C Associated with Cardio- and Myopathies on Its Interaction with Small Heat Shock Protein HspB7

**DOI:** 10.3390/ijms26125512

**Published:** 2025-06-09

**Authors:** Lydia K. Muranova, Varvara M. Vostrikova, Nikolai B. Gusev

**Affiliations:** Department of Biochemistry, School of Biology, Lomonosov Moscow State University, Moscow 119991, Russia; lidiamuranova@gmail.com (L.K.M.); varvara.vost@gmail.com (V.M.V.)

**Keywords:** filamin C, cardiomyopathies and myopathies, small heat shock proteins, HspB7

## Abstract

We investigated the interaction of HspB7 and its α-crystallin domain with the wild-type (WT) C-terminal fragment of human filamin C (FLNC), containing immunoglobulin-like domains 22–24 and its three mutants associated with cardio- and myopathies. The physicochemical properties of the WT FLNC fragment and its three mutants, p.Glu2472_Asn2473delinsAsp (EN/D) located in the 22nd domain, p.P2643_L2645del (ΔPGL), and p.W2710X (Wmut) both located in the 24th immunoglobulin-like domain were analyzed. Although all FLNC fragments had similar secondary structures, WT FLNC and its EN/D and ΔPGL mutants formed dimers, whereas Wmut formed either monomers or aggregates. The surface hydrophobicity of EN/D, ΔPGL, and especially Wmut mutants was larger than that of the WT fragment. Size exclusion chromatography, native gel electrophoresis, and chemical crosslinking indicated that the efficiency of interaction with HspB7 or its α-crystallin domain decreased in the order WT~EN/D > ΔPGL. Wmut was unable to interact with either HspB7 or its α-crystallin domain. Modeling via Alphafold 3 indicated that EN/D mutation affected the orientation of two loops connecting β-strands in the 22nd domain, while the ΔPGL and Wmut mutations exposed a hydrophobic groove in the 24th domain thereby reducing their interaction with HspB7. These findings reveal the molecular mechanisms underlying filaminopathies associated with three mutations in the C-terminal region of filamin C.

## 1. Introduction

Filamin is an important ubiquitously expressed actin-binding protein. The human genome contains three genes encoding three isoforms of filamin, the so-called filamin A (FLNA), filamin B (FLNB), and filamin C (FLNC) [1]. These isoforms are highly homologous and each monomer of filamin with a molecular weight of ~280 kDa contains two N-terminal calponin homology (CH) domains responsible for actin binding followed by 24 immunoglobulin-like domains, the last of which provides for filamin dimerization [2,3]. FLNA and FLNB are expressed ubiquitously, whereas FLNC expression is restricted to striated and cardiac muscles [2,3]. FLNB and FLNC can form heterodimers, but FLNA cannot form heterodimers with the other two filamins [4]. Although filamins have very similar primary structures, FLNC carries a unique insertion in its 20th immunoglobulin-like domain [4,5]. Filamins are multifunctional proteins interacting with more than 90 diverse cellular protein partners and playing an important role in many intracellular processes [1,6]. For instance, the best investigated FLNA serves as a versatile molecular scaffold involved in the formation of cytoskeleton and its interaction with membrane proteins, participates in integrin signaling, coordinates signaling via small GTPases [1,7], interacts with androgen receptors and affects their activity [8], and regulates phase-separated stress granule formation [9]. Less investigated FLNC plays a key role in the formation of the contractile apparatus and its interaction with the plasma membrane while also functioning as a mechanochemical sensor in skeletal and cardiac muscle [2,3,10]. FLNC is essential for myogenesis and the structural organization of Z-discs, costameres, and intercalated discs as well as for integrin signaling [6,11,12]. A wide range of FLNC mutations are associated with various cardiomyopathies including dilated, restrictive, and hypertrophic types, and myopathies collectively termed filaminopathies [11,13,14].

Small heat shock proteins (sHsps) are also among the interaction partners of FLNC. For example, the FLNC fragment comprising immunoglobulin-like domains 18–21 interacts with HspB1, and this interaction depends on HspB1 phosphorylation [15,16]. It was postulated that this interaction can play an important role in the mechanosensing properties of FLNC. Mechanical stress can induce partial FLNC denaturation leading to its aggregation. Chaperone-assisted selective autophagy (CASA) prevents the accumulation of filamin aggregation, and this process is dependent on small heat shock protein HspB8 interacting with denatured filamin and co-chaperone Bag3 [17,18]. αB-crystallin (HspB5) interacts with filamin in the Drosophila muscle and ensures structural integrity and proper muscle and cardiac performance [19].

Finally, recently published data indicate that HspB7 interacts with FLNC [20,21,22]. Earlier, we compared the interaction of the C-terminal fragment containing immunoglobulin-like domains 22–24 of filamin with four different small heat shock proteins (HspB1, HspB6, HspB8, and HspB8) and found that only HspB7 forms stable complexes with this filamin fragment [23]. HspB7 specifically interacts with the 24th immunoglobulin-like domain of FLNC and can affect its dimerization [23,24]. The α-crystallin domain of HspB7 (AcdB7) is the main site of HspB7/filamin interaction. The AcdB7 structure is different from that of the corresponding domains of the other small heat shock proteins [25]. Despite the growing body of evidence on sHsp–filamin interactions, the effects of FLNC mutations on these interactions remain insufficiently understood. Therefore, this paper deals with the analysis of the interaction of the FLNC fragment containing immunoglobulin-like domains 22–24 and its mutants associated with different forms of cardio- or myopathy with small heat shock protein HspB7 and its α-crystallin domain.

## 2. Results

### 2.1. Location of the FLNC Mutations Analyzed in This Paper

**Figure 1:**
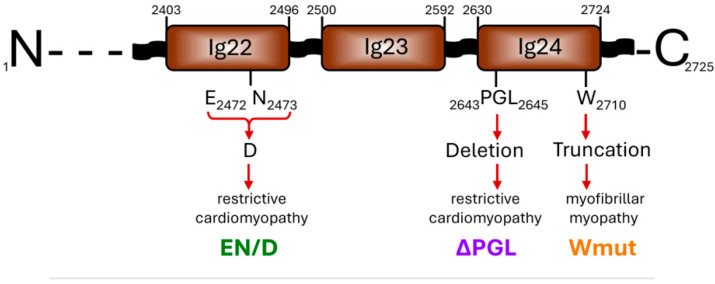
Scheme of the C-terminal end of the human FLNC structure with the location of three mutations analyzed in this paper.

We expressed and purified the C-terminal fragment of human FLNC (Uniprot Q14315) containing immunoglobulin-like domains 22–24 (amino acid residues 2403-2725) and three mutants of this construction associated with cardio- and myopathy (Figure 1). The first mutation, c.7416_7418delGAA (p.Glu2472_Asn2473delinsAsp), was located in the 22nd immunoglobulin-like domain and resulted in the replacement of Glu2472 and Asn2473 by Asp. This mutation that we will mark as EN/D is associated with restrictive cardiomyopathy [26]. The second mutation, c.7927_7935del (p.P2643_L2645del), is located in the 24th immunoglobulin-like domain and is accompanied by the deletion of tripeptide ^2643^PGL^2645^. This mutation, which we will mark ΔPGL, is also associated with restrictive cardiomyopathy [13]. Finally, the third mutation, p.W2710X, located in the 24th domain leads to the deletion of the last 16 amino acid residues. This mutation, marked as Wmut, is associated with myofibrillar myopathy [27,28].

### 2.2. Physicochemical Properties of FLNC Mutants

**Figure 2:**
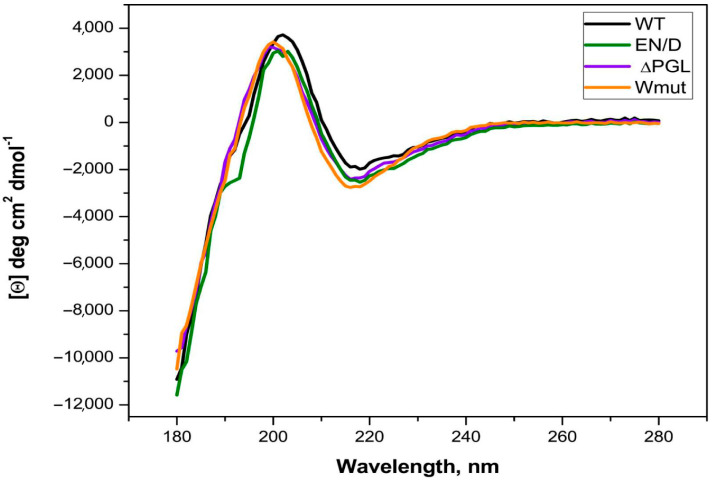
CD spectra of the wild-type FLNC fragment and its three mutants. All spectra were recorded in a 0.02 mm cell at a protein concentration of 1 mg/mL. Each measurement was repeated three times, and the results were averaged.

The wild-type (WT) FLNC fragment and its EN/D and ΔPGL mutants were easily soluble, and their purification was achieved via ion-exchange and size-exclusion chromatography. At the same time, the recombinant Wmut was poorly soluble, and 6 M urea was used for its ion-exchange purification followed by renaturation (see Section 4). In order to check the process of renaturation and to compare the secondary structure of all FLNC fragments, we recorded their CD spectra (Figure 2).

The CD spectra of all the fragments were practically indistinguishable. An estimation of the secondary structure performed by Bestsel (https://bestsel.elte.hu/index.php, accessed on 17 April 2025) indicated that the secondary structure of all proteins consisted of about 42–47% antiparallel β-sheets, 11–13% turns, and 0% α-helices. These data correlate with the expected FLNC fragment structure and indicate that the secondary structures of all the fragments are comparable and the renaturation of Wmut was successful. In agreement with this statement, at an ionic strength of more than 50 mM, the solubility of all FLNC fragments was similar.

**Figure 3:**
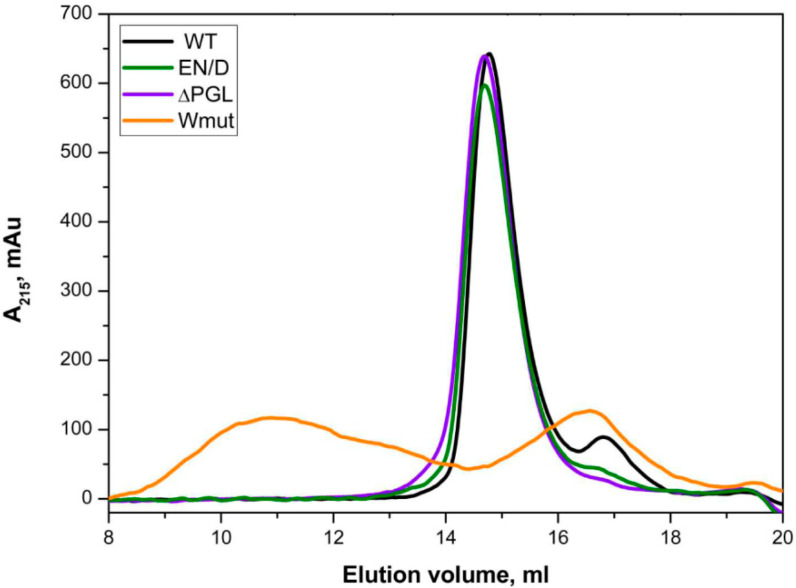
Size-exclusion chromatography of FLNC fragments. Equal quantities of FLNC fragments (0.1 mg) dissolved in 100 µL of buffer were loaded on a Superdex 200 10/300 column and eluted at a rate of 0.5 mL/min.

Size-exclusion chromatography was used for the analysis of the oligomeric structure of all FLNC fragments. As indicated in Figure 3, all proteins except Wmut were eluted as symmetrical sharp peaks with an apparent molecular weight of ~80 kDa. At the same time, Wmut formed two peaks, one broad peak with an apparent molecular weight of ~580 kDa and another small peak with an apparent molecular weight of ~35 kDa.

The data presented indicate that the WT FLNC fragment and its EN/D and ΔPGL mutants form dimers, whereas Wmut forms monomers with an apparent molecular weight of ~35 kDa and aggregates (or oligomers) with an apparent molecular weight of ~580 kDa.

**Figure 4:**
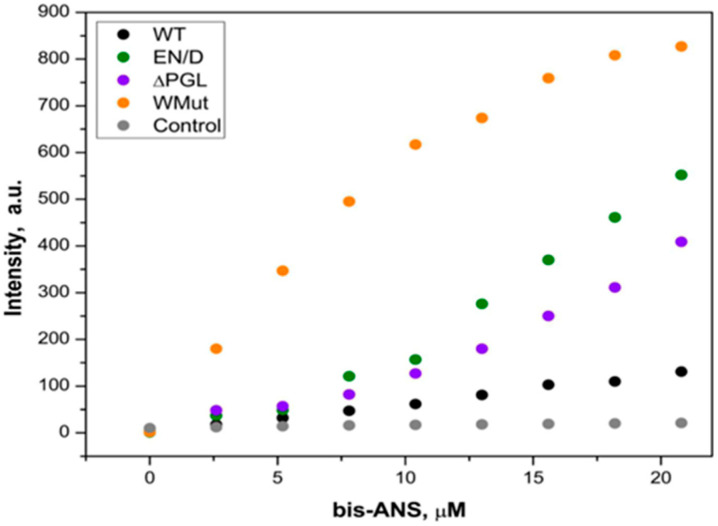
Fluorescent titration of different FLNC fragments (0.10 mg/mL) with bis-ANS. Fluorescence was excited at 395 nm and recorded at 500 nm. In the control experiment, incubation buffer without addition of any FLNC fragments was titrated with bis-ANS. Representative data of four independent experiments are shown.

In order to further characterize the structural properties of filamin fragments, we analyzed their surface hydrophobicity. We titrated filamin fragments with the hydrophobic probe, bis-ANS, and recorded the fluorescence of the complex formed by these proteins and the fluorescent probe. As indicated in Figure 4, the titration of the WT FLNC fragment by using bis-ANS was accompanied by only a marginal increase in fluorescence. At the same time, the titration of the EN/D, ΔPGL, and especially Wmut was accompanied by a significant increase in fluorescence (Figure 4). These data mean that the surface hydrophobicity of FLNC fragments decreases in the order Wmut > ΔPGL~EN/D > WT.

It is worthwhile mentioning that at a low ionic strength, any contacts of Wmut with glass or quartz were accompanied by rapid protein aggregation. This aggregation can be prevented by the addition of NaCl (up to 50 mM), or 10% glycerol or sucrose. In order to prevent aggregation, all solutions of FLNC Wmut in low ionic strength buffers were kept in plastic.

### 2.3. Interaction of FLNC Fragments with HspB7 and Its α-Crystallin Domains Analyzed via Size-Exclusion Chromatography

**Figure 5:**
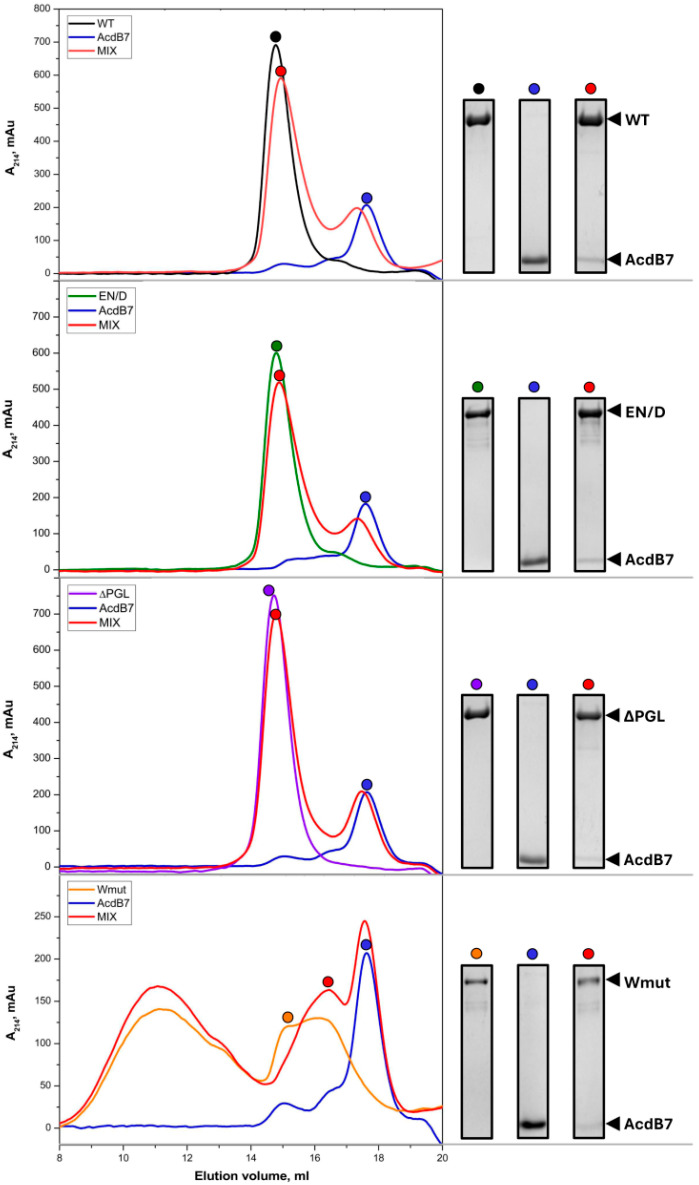
Size-exclusion chromatography of FLNC fragments, AcdB7, and their mixtures. One hundred microliters of isolated AcdB7 (30 µM) (blue line), isolated FLNC fragments (30 µM) (green, blue, or yellow lines), or their mixture (red line) were loaded on a Superdex 200 10/300 column and eluted at a rate of 0.5 mL/min. The protein composition of peak fractions marked by differently colored dots was analyzed via SDS-PAAG. Each experiment was repeated three times.

We previously demonstrated that the main sites of HspB7-FLNC interaction are located in the α-crystallin domain of HspB7 (AcdB7). This domain specifically and tightly interacts with the WT FLNC fragment, and this interaction can be effectively monitored by size-exclusion chromatography [23]. Using this method, we analyzed the interaction of AcdB7 with all FLNC mutants (Figure 5).

As indicated in Figure 5, mixing wild-type (WT) FLNC and its EN/D mutant with AcdB7 resulted in a shift of the AcdB7 peak toward lower elution volumes, accompanied by the appearance of AcdB7 within the FLNC fragment peak. Similar but smaller changes in the elution profile were detected in the case of the mixture of ΔPGL FLNC and AcdB7. The elution profile of the mixture of FLNC Wmut and AcdB7 was close to the sum of elution profiles of the isolated FLNC fragment and AcdB7, and we were unable to detect any significant quantities of AcdB7 in the peaks of FLNC Wmut. The data presented indicate that both WT FLNC and its EN/D and ΔPGL mutants interact with AcdB7, whereas FLNC Wmut shows little to no interaction with AcdB7.

Qualitatively similar results were obtained when the full-size HspB7 was mixed with FLNC fragments (Figure S1). Due to the comparable apparent molecular weights of FLNC fragments and full-length HspB7, their peaks were not fully resolved via size-exclusion chromatography using either Superdex 75 or Superdex 200. However, even in this case, the mixing of WT FLNC or its EN/D mutant with HspB7 was accompanied by a decrease in the peak corresponding to isolated HspB7 and shifting of HspB7 toward the peak of the FLNC fragment. These changes in the elution profile were less pronounced in the case of the ΔPGL FLNC mutant (Figure S1). As mentioned earlier (Figure 3), FLNC Wmut was eluted on Superdex 200 in the form of two peaks. This makes it difficult to analyze its interaction with full-size HspB7. However, the mixing of FLNC Wmut with full-size HspB7 was not accompanied by any significant changes in the distribution of Wmut or HspB7 on the elution profile. Thus, as in the case with AcdB7, the efficiency of interaction with FLNC fragments decreased in the order WT~EN/D > ΔPGL, and FLNC Wmut did not interact with the full-size HspB7.

### 2.4. Analysis of the Interaction of Mutant Filamin Fragments with HspB7 via Native Gel Electrophoresis

**Figure 6:**
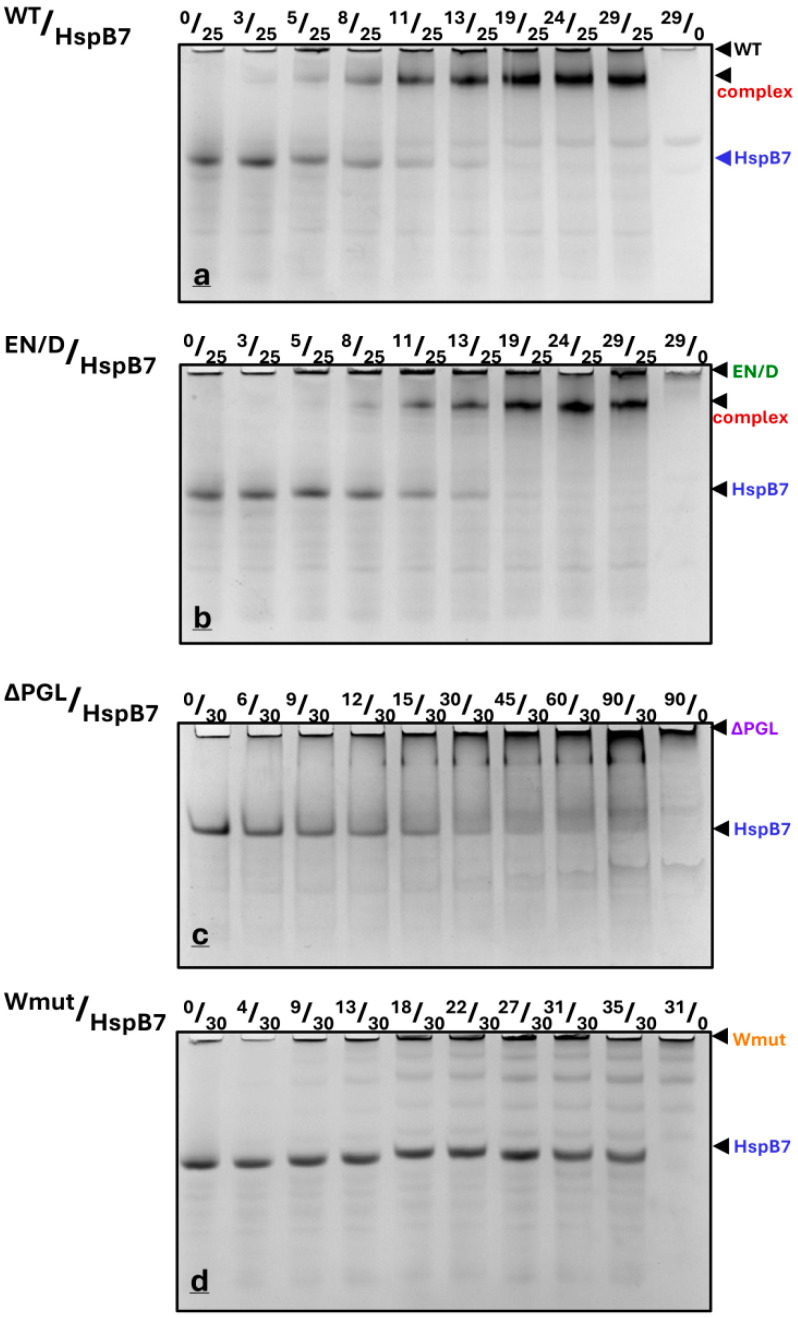
Fixed quantities of HspB7 (25 or 30 µM) were mixed with different quantities of FLNC fragments (their nature is indicated above each panel), incubated for 30 min at 30 °C, and subjected to native gel electrophoresis. The molar ratio of FLNC fragments and HspB7 (in μM) is indicated above each track. Representative data of four independent experiments are shown. The positions of isolated HspB7, isolated FLNC fragments, and their complex are marked by arrows.

All filamin fragments have alkaline pI, whereas HspB7 and its α-crystallin domain have an acidic isoelectric point. This makes it possible to use native gel electrophoresis at pH 8.6 for the analysis of the interaction of these proteins. Under these conditions, filamin fragments have low electrophoretic mobility and do not enter the gel. In contrast, HspB7 and its α-crystallin domain have high electrophoretic mobility and any complexes of FLNC fragments and HspB7 (AcdB7) have intermediate electrophoretic mobility. As previously demonstrated, we have shown that this interaction is not a result of electrostatic interaction and is highly specific [23]. The titration of fixed quantities of HspB7 (25–30 µM) by using WT FLNC or its EN/D mutant was accompanied by a gradual decrease in the band corresponding to isolated HspB7 and a parallel increase in the band located on the top of the gel and probably corresponding to the complex formed by HspB7 and filamin fragments (Figure 6a,b). The titration of HspB7 by using ΔPGL resulted only in a marginal decrease in the band corresponding to isolated HspB7 and the formation of only a very small diffuse band on the top of the gel probably corresponding to the complex (Figure 6c). Finally, the addition of even very large quantities of Wmut to HspB7 had no effect on the intensity of the band corresponding to isolated HspB7 and the appearance of any bands corresponding to the complex formed by these proteins (Figure 6d).

We determined the integral intensity of the bands corresponding to isolated HspB7 and its complex formed with WT FLNC and the EN/D mutant of FLNC (Figure S2). Both in the cases of WT FLNC and its EN/D mutant, a half-maximal decrease in the intensity of the band of isolated HspB7 and a half-maximal increase in the complex formation occurred roughly at a similar concentration of FLNC added (~10 µM), thus indicating that these two fragments of filamin interact with HspB7 with similar apparent affinity. In both cases, the complete disappearance of the band corresponding to isolated HspB7 and the saturation of the band corresponding to the complex formed was detected at the FLNC fragment concentration that was roughly two times smaller than that of HspB7, thus indicating the formation of the HspB7/FLNC complex with stoichiometry equal to 2/1 (Figure S2). Although the data presented are semi quantitative, they clearly indicate the difference in the interaction of HspB7 with four different filamin fragments. Future experiments with the utilization of modern biophysical techniques like isothermal titration calorimentry or surface plasmon resonance will provide additional quantitative information on HspB7/FLNC fragment interaction.

**Figure 7:**
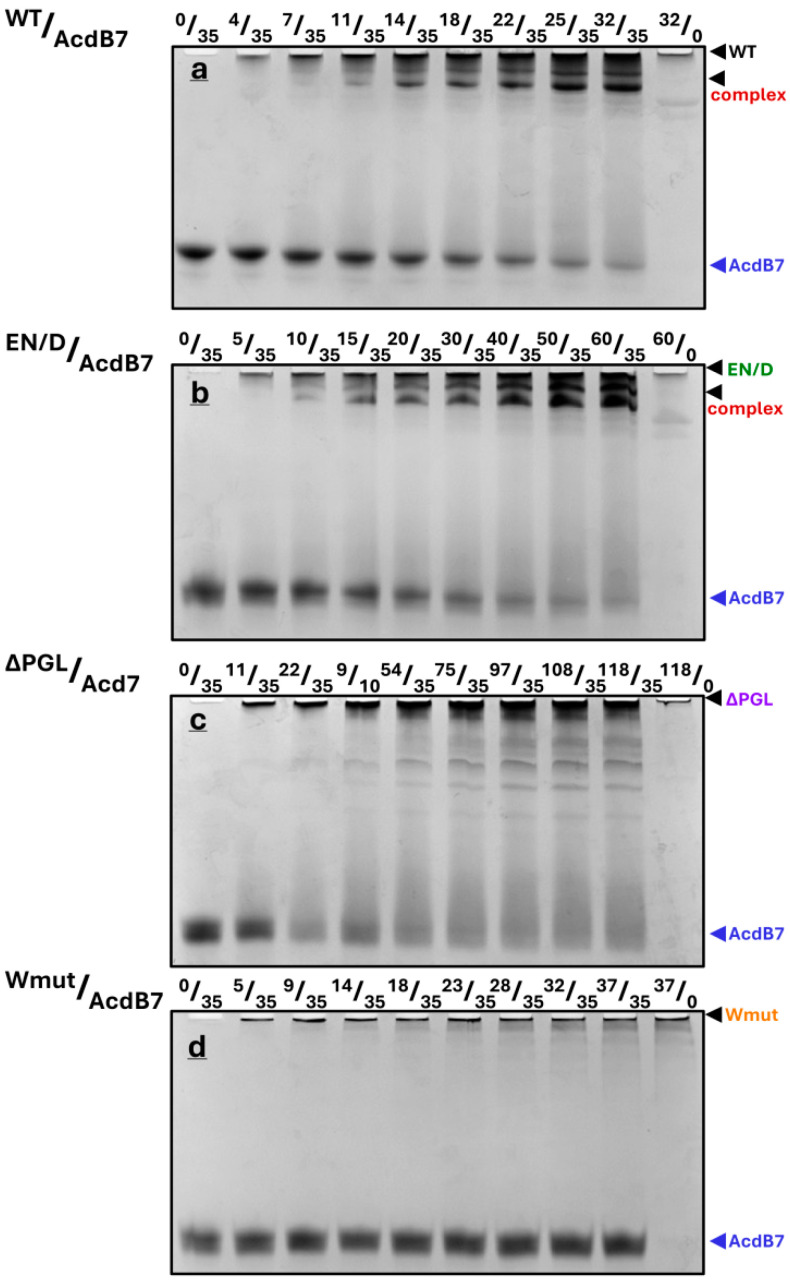
Fixed quantities of AcdB7 (35 µM) were mixed with different quantities of FLNC fragments (their nature is indicated above each panel), incubated at 30 °C for 30 min, and subjected to native gel electrophoresis. The molar ratio of FLNC fragments and AcdB7 (in µM) is indicated above each track. Representative data of three independent experiments are shown. The positions of isolated AcdB7, FLNC fragments, and their complex are marked by arrows.

Similar experiments were run with AcdB7 and different fragments of filamin (Figure 7). As in the case of the full-size HspB7, the titration of the α-crystallin domain of HspB7 by using WT FLNC or its EN/D mutant resulted in a gradual decrease in the band corresponding to AcdB7 and the appearance of one or two bands on the top of the gel, which probably correspond to the complexes formed (Figure 7a,b). In the case of the ΔPGL mutant, the mixing with AcdB7 was accompanied by the smearing of the band of AcdB7 and the formation of a weak diffuse band corresponding to the complex on the top of the gel (Figure 7c). The Wmut of FLNC was unable to interact with AcdB7 (Figure 7d).

Under the conditions used, a half-maximal decrease in the band of AcdB7 was observed at a concentration of WT FLNC or its EN/D mutant equal to ~20 µM, and the disappearance of the band corresponding to isolated AcdB7 was observed at a concentration of FLNC fragments equal to that of AcdB7, thus indicating the formation of the complex with stoichiometry of 1/1 (Figure S3).

### 2.5. Crosslinking of Mutant Filamin Fragments with HspB7

**Figure 8:**
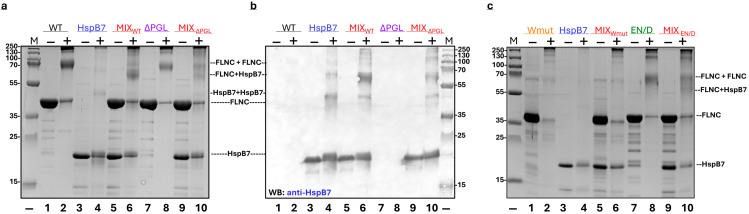
Crosslinking of HspB7 with different FLNC fragments. (**a**) Crosslinking of WT FLNC and its ΔPGL with HspB7. Isolated WT FLNC (tracks 1, 2), isolated ΔPGL mutant (tracks 7, 8), isolated HspB7 (tracks 3, 4), or equimolar mixtures of WT FLNC and HspB7 (tracks 5, 6) and the equimolar mixture of ΔPGL and HspB7 (tracks 9, 10) were run on SDS gel electrophoresis. Proteins were preincubated either in the absence (−) or in the presence (+) of glutaraldehyde (GA). The positions of crosslinked complexes and molecular mass markers are indicated by arrows. (**b**) Immunoblot of the above-indicated gel stained with anti-HspB7 antibodies. (**c**) Crosslinking of Wmut and EN/D mutant of FLNC with HspB7. Isolated Wmut (tracks 1, 2), isolated EN/D mutant (tracks 7, 8), isolated HspB7 (tracks 3, 4), and an equimolar mixture of HspB7 and Wmut (tracks 5, 6) or EN/D mutant (tracks 9, 10) were run on SDS gel electrophoresis. Proteins were preincubated either in the absence (−) or in the presence (+) of GA. The positions of crosslinked complexes and molecular mass markers are indicated by arrows. Representative data of three independent experiments are shown.

The method of chemical crosslinking was also used for the analysis of the interaction between HspB7 and its α-crystallin domain with different mutant filamin fragments. Crosslinking of WT FLNC resulted in a decrease in the band corresponding to the WT FLNC monomer and accumulation of a new band with an apparent molecular weight of ~70 kDa probably corresponding to the crosslinked dimer of WT FLNC (Figure 8a). In addition, high-molecular-weight crosslinked complexes were also detected on the gel. Isolated HspB7 was not practically crosslinked by using glutaraldehyde (GA) (Figure 8a). Crosslinking of the mixture of HspB7 and WT FLNC resulted in the appearance of a new band with an apparent molecular weight of ~58 kDa and a decrease in the band corresponding to the FLNC dimer. Data of immunoblotting (Figure 8b) indicate that this new band contains HspB7, thus indicating crosslinking of WT FLNC with HspB7. Similar experiments were performed with ΔPGL and EN/D mutants of FLNC (Figure 8b,c). In both cases, crosslinking with glutaraldehyde resulted in the formation of the same protein band with an apparent molecular weight of ~58 kDa, although the efficiency of crosslinking was slightly lower than in the case of WT FLNC (Figure 8c). Crosslinking of the Wmut of FLNC with glutaraldehyde resulted in the accumulation of only a very-high-molecular-weight crosslinked product and practically complete disappearance of the band corresponding to the Wmut of FLNC (Figure 8c). We were unable to detect any additional bands after the crosslinking of the Wmut of FLNC and HspB7, thus indicating that this fragment of filamin cannot be crosslinked to HspB7.

Similar results were obtained if the α-crystallin domain of HspB7 was crosslinked with different FLNC fragments (Figure S4). The crosslinked product formed in the case of WT FLNC and AcdB7 had an apparent molecular weight of ~45 kDa. Similar products were detected in the case of crosslinking of AcdB7 with ΔPGL and EN/D mutants of FLNC (Figure S4), although the efficiency of crosslinking these fragments with AcdB7 was lower than in the case of WT FLNC. The Wmut of FLNC was not crosslinked to AcdB7 (Figure S4).

## 3. Discussion

In this paper, we analyze some properties of three fragments of FLNC containing C-terminal 22nd–24th immunoglobulin-like domains. Mutation EN/D located in the 22nd immunoglobulin domain and associated with restrictive cardiomyopathy [26] had no effect either on the oligomeric structure of the FLNC fragment or on its secondary structure (Figure 2 and Figure 3). However, the surface hydrophobicity of the EN/D mutant was larger than that of the WT fragment of FLNC (Figure 4). Mutation ΔPGL leads to the deletion of tripeptide PGL in the structure of the 24th immunoglobulin-like domain of FLNC and is associated with restrictive cardiomyopathy [13]. The secondary structure of the ΔPGL mutant was similar to that of the WT fragment (Figure 2), and it formed dimers as the WT fragment (Figure 3). At the same time, this mutant interacted with the fluorescent probe bis-ANS more efficiently than the WT protein (Figure 4). This means that the surface hydrophobicity of this mutant is larger than that of the WT protein. The mutation Wmut results in the deletion of the last 16 amino acid residues of FLNC and is associated with myofibrillar myopathy [27,28,29]. It was reported that this mutation led to dramatic changes in the CD spectrum of the isolated 24th domain of FLNC [29]. In our case of a much larger fragment containing the 22nd–24th domains of FLNC, this mutation had no significant effect on the CD spectrum (Figure 2). Each immunoglobulin-like domain of FLNC contains from six up to eight β-strands. This means that our large FLNC fragment contains at least 18 large β-strands, whereas the isolated 24th domain contains only 7 β-strands. Therefore, the deletion of the last β-strand formed by the C-terminal amino acid residues will induce pronounced changes in the secondary structure of the short fragment containing the isolated 24th domain and will induce only minimal changes in the structure of the large fragment containing three C-terminal immunoglobulin-like domains of FLNC. As already mentioned, the WT FLNC fragment formed stable dimers. In contrast, in size-exclusion chromatography, Wmut was present in the form of two peaks corresponding, according to their apparent molecular weight, to monomers and large-size aggregates (or oligomers) (Figure 3). This finding agrees with the data of the literature indicating that the full-size FLNC W2710X mutant and its 24th mutant domain were unable to form stable dimers and tended to aggregate [21,27,28,29]. Aggregation can be at least partially due to the increased surface hydrophobicity of Wmut, which was detected via its efficient interaction with the fluorescent probe bis-ANS (Figure 4).

Size-exclusion chromatography, native gel electrophoresis, and chemical crosslinking were used to analyze the interaction of different fragments of FLNC with HspB7 and its α-crystallin domain. The data of SEC indicate that all fragments of FLNC except Wmut interact with HspB7 (AcdB7). This interaction was accompanied by the shifting of the peak corresponding to HspB7 (or AcdB7) toward smaller elution volumes and the appearance of sHsps in the peak corresponding to FLNC fragments (Figure 5 and Figure S1). We were unable to detect the interaction of Wmut with HspB7 (or AcdB7) by means of SEC. Although SEC can be used for the analysis of the interaction between FLNC fragments and HspB7, this method has certain limitations.

The data of native gel electrophoresis indicate that the titration of fixed quantities of HspB7 (or AcdB7) by increasing the quantities of WT FLNC or its EN/D mutant was accompanied by the complete disappearance of the band corresponding to HspB7 (or AcdB7) and the accumulation of the band corresponding to the complex formed between these two proteins (Figure 6 and Figure 7). The stoichiometry of complexes formed was close to 1:1 in the case of FLNC fragments and AcdB7 and close to 2:1 in the case of FLNC fragments and full-size HspB7. This difference in stoichiometry can be explained by the suggestion that FLNC fragments interact with the dimer of HspB7 and with AcdB7, which is predominantly presented as a monomer [25]. Alternatively, one cannot exclude the presence of two HspB7 binding sites on FLNC fragments. One of these sites possessing high affinity is occupied by both full-size HspB7 and its AcdB7, whereas the second site with lower affinity can be occupied only by full-size HspB7. Under the conditions used, the titration of fixed quantities of HspB7 (or AcdB7) by the ΔPGL mutant resulted in an incomplete decrease in the band corresponding to isolated sHsps and lack of accumulation of the band corresponding to the complex formed by these proteins (Figure 6 and Figure 7). By using native gel electrophoresis, we were unable to detect any interaction of Wmut with HspB7 or AcdB7. Thus, in accordance with the data of SEC, the efficiency of interaction between FLNC fragments and HspB7 (or AcdB7) decreases in the order WT~EN/D> ΔPGL, whereas Wmut was unable to interact with HspB7 (or AcdB7).

Similar results were obtained in experiments with chemical crosslinking. Indeed, in good agreement with our earlier published data [23], the incubation of WT FLNC and HspB7 with glutaraldehyde resulted in the formation of a crosslinked complex with an apparent molecular weight of ~58 kDa, which was stained by using anti-HspB7 antibodies (Figure 8). The apparent molecular weight of this complex correlates with the sum of the apparent weights of the monomer of HspB7 and the monomer of the FLNC fragment. The crosslinking of EN/D and ΔPGL mutants with HspB7 also resulted in the formation of a similar complex; however, the intensity of the band corresponding to the crosslinked complex was slightly smaller than in the case of the WT fragment of FLNC (Figure 8). Crosslinking of any isolated FLNC fragment except Wmut resulted in the accumulation of the band with an apparent molecular weight of ~70 kDa corresponding to the dimer. The incubation of Wmut with glutaraldehyde resulted in the accumulation of only high-molecular-weight oligomers without the appearance of the band corresponding to the dimer (Figure 8). These data agree with the data of size-exclusion chromatography (Figure 3) and the previously published results [29] and indicate that Wmut is unable to form dimers and is present in the form of monomers tending to aggregate. Similar results were obtained if FLNC fragments were crosslinked with AcdB7 (Figure S4). A crosslinked complex with an apparent molecular weight of ~45 kDa was detected in the case of all analyzed FLNC fragments except Wmut, which was not crosslinked to AcdB7 (Figure S4). The apparent molecular weight of 45 kDa agrees well with the sum of apparent molecular weights of AcdB7 and the FLNC fragment, and the efficiency of crosslinking decreased in the order WT~EN/D > ΔPGL.

The question arises as to why the analyzed mutation can affect the interaction of FLNC fragments with HspB7 or its α-crystallin domain. The EN/D mutation is located in the 22nd domain, i.e., far from the 24th domain where the main site of FLNC interaction with HspB7 is located. Probably, therefore, this mutation only weakly affects FLNC–HspB7 interaction. A small negative effect of this mutation on the FLNC/HspB7 interaction can be explained by the structural changes in the 22nd immunoglobulin-like domain or interaction of this domain with two other C-terminal domains of FLNC. Modeling using Alphafold 3 indicates that in the WT protein, Glu2472 forms a hydrogen bond with Ser2448, thus fixing two loops connecting CD and FG β-strands belonging to two different β-sheets (Figure S5). The EN/D mutation prevents this interaction destabilizing these β-sheets and probably disclosing certain hydrophobic sites as it was detected in experiments with bis-ANS titration (Figure 4). These structural changes can weakly affect the interaction of the EN/D mutant with HspB7 or AcdB7.

**Figure 9:**
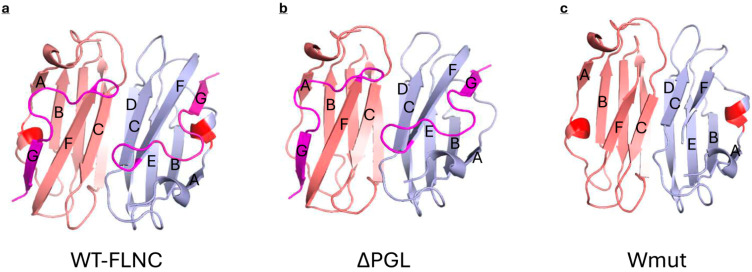
Structure of the 24th domain of filamin C determined via X-ray crystallography (**a**) and models of the structure of the 24th domain of ΔPGL (**b**) (ipTM 0.75, pTM 0.78) and Wmut (**c**) (ipTM 0.69, pTM 0.72) mutants of FLNC predicted by Alphafold 3. Two monomers are marked light blue and salmon. β-strands are marked by letters, and the C-terminal G strand and the loop connecting strands F and G are marked magenta. Mutated tripeptide PGL is marked red.

Two other mutations (ΔPGL and Wmut) are located in the 24th domain and therefore can directly affect the interaction of FLNC with HspB7. The 24th domain of FLNC contains two β-sheets. One sheet is formed by strands A, B, E, and D, and another sheet is formed by strands C, F, and G (Figure 9) [30]. Tripeptide PGL is included in the short 3/10-helix located in the loop connecting strands A and B (Figure 9) [30,31]. This tripeptide is highly conserved and is detected in the primary structure of human, rat, mouse, and chicken FLNC [30]. Moreover, similar tripeptides (LGL or AGL) are detected in the primary structure of human, rat, mouse, rabbit, and chicken filamin A and filamin B [30]. The loop connecting strands A and B forms a hydrogen bond with the end of strand G [30]. Strand G belongs to the sheet that is formed by strands C, F, and G, and therefore, the deletion of tripeptide PGL can indirectly affect the orientation of strand C, playing a crucial role in the dimerization of the 24th domain and its interaction with HspB7. Moreover, PGL tripeptide covers the hydrophobic groove formed by strands A, B, and F. PGL deletion will leave this groove open and will increase the surface hydrophobicity of the ΔPGL mutant. This effect was detected in experiments with the fluorescent probe bis-ANS (Figure 4). The indirect effect of PGL on the orientation of strand C playing a crucial role in the interaction of AcdB7 with the 24th domain of FLNC can also affect the binding of HspB7 to the ΔPGL mutant of FLNC. Indeed, ΔPGL weakly interacts with HspB7 or AcdB7.

Even larger conformational changes are induced by the deletion of the last 16 amino acid residues in the Wmut fragment of FLNC. Modeling with Alfafold 3 (Figure 9) indicates that the deletion of the last G strand results in a decrease in the length of strands C and F. This will destabilize the interface of the monomer–monomer interaction and the overall structure of the 24th domain. In addition, G strands (like PGL tripeptide) cover the hydrophobic groove formed by strands A, B, and F. The deletion of strand G leaves this groove unprotected and should increase the hydrophobicity of the Wmut of FLNC. Indeed, as indicated in Figure 4, Wmut has the highest surface hydrophobicity among the analyzed FLNC fragments. Therefore, Wmut cannot form stable dimers and tends to aggregate as it was detected by means of size-exclusion chromatography (Figure 3). Dramatic destabilization and tending to aggregate prevent the effective interaction of Wmut with HspB7 or AcdB7.

We hope that our data will be helpful for understanding the molecular mechanisms underlying the effect of other earlier described mutations in the 24th immunoglobulin-like domain of FNC associated with myo- and cardiomyopathies [11,32,33]. Moreover, the recently published data indicate that the detailed proteomic analysis of aggregates formed by FLNC with mutations in the 24th immunoglobulin-like domain can be used for the verification of new filaminopathy biomarkers [33]; therefore, the analysis of filamin interaction with protein partners seems especially important.

Thus, the data presented uncover the molecular mechanisms underlying some filaminopathies associated with mutations in the 24th immunoglobulin-like domain playing an important role in filamin dimerization and its interaction with HspB7.

## 4. Materials and Methods

### 4.1. Proteins

The WT FLNC fragment and its EN/D and ΔPGL mutants were expressed and purified as described earlier [23]. The Wmut FLNC fragment tends to aggregate and was accumulated in inclusion bodies. Bacteria Rosetta 2 (DE3)pLysS expressing this mutant were suspended in buffer A (20 mM phosphate buffer pH 7.0 containing 15 mM β-ME, 0.1 PMSF), subjected to lysozyme and benzonase treatment, and sonicated, and inclusion bodies were collected via centrifugation. Inclusion bodies were washed three times with buffer A and dissolved in the same buffer containing 6 M urea. After incubation for 60 min, the solution obtained was subjected to ultracentrifugation (105.000× *g*, 30 min). The supernatant was loaded on a 5 mL HiTrap SP column equilibrated with buffer A containing 6 M urea. The column was washed by using 5 column volumes of buffer A containing 6 M urea, and proteins were eluted with 8 column volumes of linear gradient formed by using buffer A containing 6 M urea and buffer B containing 20 mM phosphate buffer (pH 7.0), 15 mM β-ME, 0.1 PMSF, 1 M urea, and 0.4 M NaCl. Fractions enriched with Wmut FLNC were collected and dialyzed against buffer C (20 mM Tris-acetate pH 7.6, 10 mM NaCl, 2 mM DTT, 0.1 mM EDTA, 0.1 mM PMSF). After centrifugation, all the FLNC fragments were aliquoted and stored at −80 °C. The concentration of all FLNC fragments was determined spectrophotometrically using A^0.1%^_280_ equal to 0.6.

HspB7 and its α-crystallin domain (AcdB7) containing residues 77–155 were expressed and purified as described earlier [25,34]. The concentration of AcdB7 was determined spectrophotometrically A ^0.1%^_280_ equal to 0.161. The concentration of HspB7 was determined using Ponceau as described earlier [35] using bovine serum albumin as a standard.

### 4.2. CD Spectroscopy

All proteins (1 mg/mL) were dialyzed against buffer D (30 mM sodium phosphate pH 7.4 containing 150 mM NaF) and subjected to centrifugation. CD spectra were recorded on a Chirascan CD spectrometer in the range of 180–280 nm at a rate of 20 nm/min at 10 °C in a 0.02 mm cell. Each measurement was repeated three times, and the results were averaged. An estimation of the different elements of the secondary structure was performed using the Bestsel program (https://bestsel.elte.hu/index.php, accessed on 17 April 2025).

### 4.3. Fluorescence Spectroscopy

All measurements were performed in buffer E (20 mM HEPES/NaOH pH 7.4, containing 150 mM NaCl, 0,1 mM EDTA, 15 mM β-ME) at 25 °C on a Varian Cary Eclipse spectrofluorimeter (Agilent, Santa Clara, CA, USA). Proteins (0.1 mg/mL) were titrated with bis-ANS so that the final concentration of the fluorescent probe varied between 0 and 20 µM. In the control experiment, buffer E without the addition of any FLNC fragments was titrated with bis-ANS. Fluorescence was excited at 395 nm (slit with 5 nm) and recorded at 500 nm (slit width 10 nm).

### 4.4. Size-Exclusion Chromatography

Size-exclusion chromatography was performed on a Superdex 200 HR10/300 column (Marlborough, MA, USA) equilibrated with buffer F (50 mM Na-phosphate pH 7.4, containing 150 mM NaCl, 0.1mM EDTA, 0.1 mM PMSF, 2 mM DTT) at room temperature. Proteins (50–100 µL) were loaded on the column and eluted at a rate of 0.5 mL/min, and the elution profile was recorded at 214 nm. Samples (400 µL) were collected, mixed with 50 µL of trichloroacetic acid, and subjected to centrifugation. The protein pellet was washed with acetone, and its composition was determined via SDS gel electrophoresis [36]. For analyzing protein–protein interaction, isolated proteins or their equimolar mixture were preincubated for 30 min at 30 °C and subjected to centrifugation before loading on the column. The column was calibrated with thyroglobulin (669 kDa), ferritin (440 kDa), rabbit muscle glyceraldehyde-3-phosphate dehydrogenase (144 kDa), ovotransferrin (77 kDa), ovalbumin (43 kDa), and carboanhydrase (29 kDa).

### 4.5. Native Gel Electrophoresis

Fixed quantities of HspB7 or AcdB7 (usually 25–30 µM) in buffer C, containing 100 mM NaCl, were mixed with different quantities (0–120 µM) of FLNC fragments and incubated for 30 min at 30 °C. Thus, the obtained probes (40 µL) were mixed with 10 µL of sample buffer (40 mM Tris-glycine, pH 8.6, 25% glycerol, 4% β-ME) and loaded on native 12.5% polyacrylamide gel containing 10% glycerol. Electrophoresis was performed in 80 mM Tris-glycine buffer pH 8.6 [37]. After staining with Coomassie R250, the gels were evaluated using the Gel Analyzer 23.1 program.

### 4.6. Chemical Crosslinking with Glutaraldehyde (GA)

Isolated FLNC fragments, isolated HspB7 (or AcdB7), or their equimolar mixture were incubated for 30 min at 30 °C in 25 mM HEPES/NaOH (pH 7.4) containing 100 mM NaCl and 2 mM DTT. Equal volumes of water or GA solution (final concentration 0.0125%) were added to the protein samples, and the incubation was continued for another 30 min at 30 °C. The reaction was quenched by adding the SDS sample buffer, and the proteins were subjected to SDS gel electrophoresis [36].

After gel electrophoresis, they were immunoblotted in modified Dunn carbonate buffer (10 mM NaHCO_3_, 3 mM Na_2_CO_3_, pH 9.9 containing 10% ethanol). After blocking with 5% non-fat milk in TBST buffer (20 mM Tris, 0.15 M NaCl, 0.1% Tween20), the blot was incubated with primary mouse monoclonal anti-HspB7 antibodies (6E1) kindly provided by Dr. A.G. Katrukha (Department of Biochemistry, School of Biology, Moscow State University) followed by washing and incubation with secondary antibodies conjugated with horseradish peroxidase. Protein bands were detected by using the Invitrogen chemiluminescence commercial kit.

## Data Availability

Data is contained within the article or Supplementary Materials.

## Supplementary Materials

The following supporting information can be downloaded at: https://www.mdpi.com/article/10.3390/ijms26125512/s1.
