# Supplementary material for: Effect of Mutations in the C-Terminal 22–24 Domains of Filamin C Associated with Cardio- and Myopathies on Its Interaction with Small Heat Shock Protein HspB7
Source: Int J Mol Sci. 2025 Jun 9;26(12):5512. doi: 10.3390/ijms26125512 (PMC12193154; doi:10.3390/ijms26125512)
Supplement: Supplementary file 1 [file ijms-26-05512-s001.zip › ijms-3660425-supplementary.pdf]

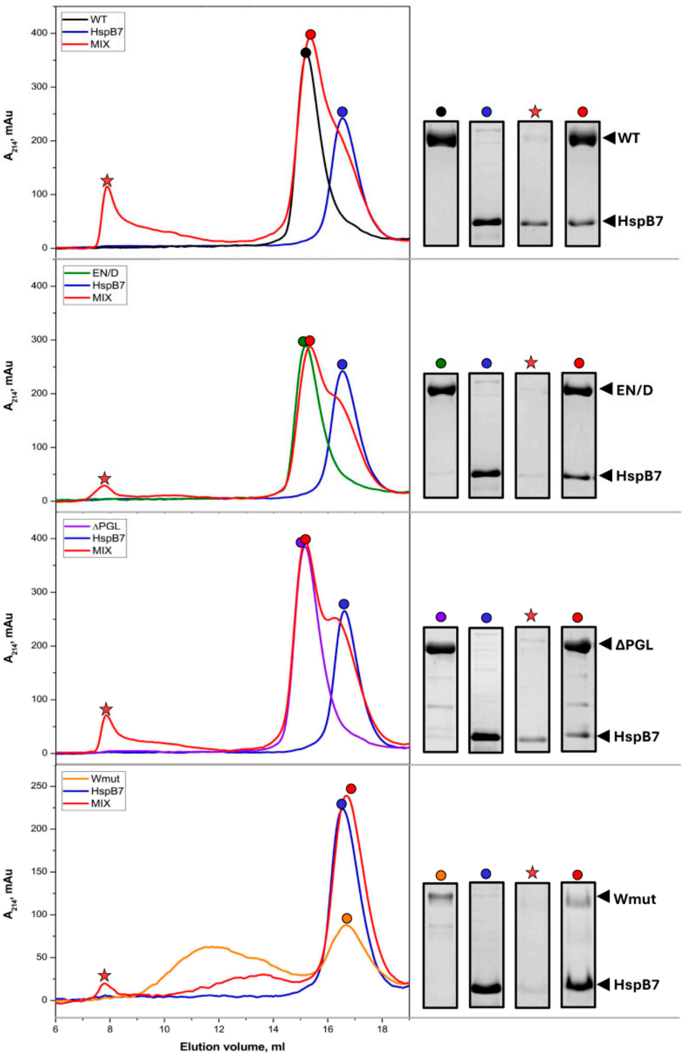

Figure S1. Size-exclusion chromatography of FLNC fragments, HspB7, and their mixtures. One hundred microliters of isolated AcdB7 (30 μM) (blue line), isolated FLNC fragments (30 μM) (green, blue, or yellow lines), or their mixture (red line) were loaded on a Superdex 200 10/300 column and eluted at a rate of 0.5 ml/min. The protein composition of peak fractions marked by differently colored dots was analyzed by SDS-PAAG.

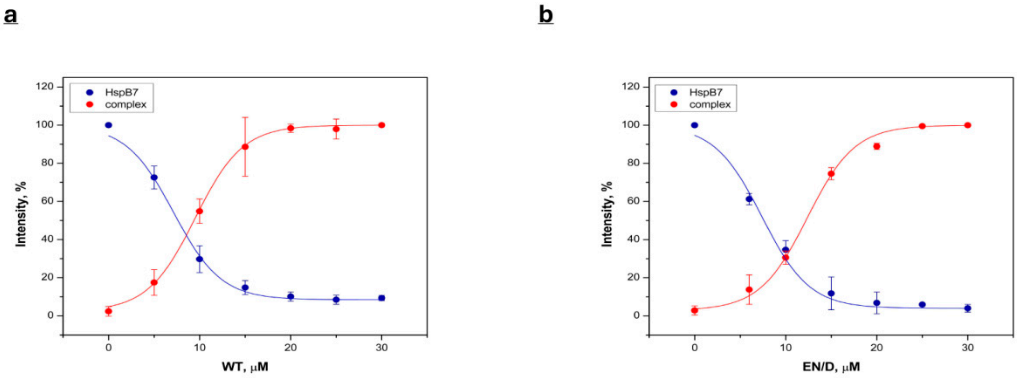

Figure S2. Dependence of the intensity of the band of isolated HspB7 (blue line) and of the band of the complex formed (red line) on FLNC WT (a) or EN/D fragment (b) concentration. Average data from four independent experiments are presented.

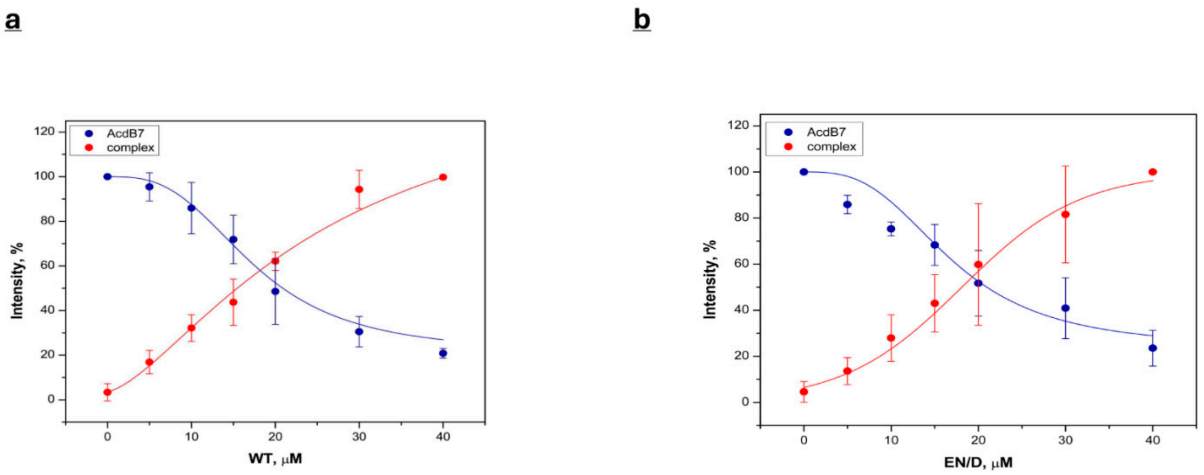

Figure S3. Dependence of the intensity of the band of isolated AcdB7 (blue lines) and of the band of the complex formed (red lines) on FLNC WT (a) or EN/D fragment (b) concentration. Average data from three independent experiments are presented.

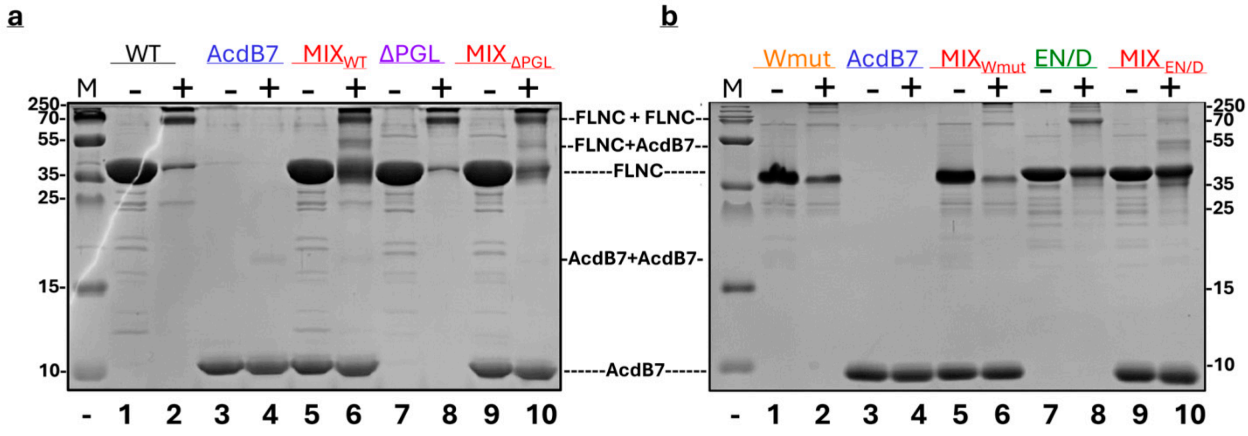

Figure S4. Crosslinking of AcdB7 with different FLNC fragments. a). Crosslinking of WT FLNC and its ΔPGL with AcdB7. Isolated WT FLNC (tracks 1, 2), isolated ΔPGL mutant (tracks 7, 8), isolated AcdB7 (tracks 3, 4), or equimolar mixtures of WT FLNC and AcdB7 (tracks 5, 6) and the equimolar mixture of ΔPGL and AcdB7 (tracks 9, 10) were run on the SDS-gel electrophoresis. Proteins were preincubated either in the absence (-) or in the presence (+) of glutaraldehyde (GA). The positions of crosslinked complexes and molecular mass markers are indicated by arrows. b). Crosslinking of Wmut and EN/D mutant of FLNC with AcdB7. Isolated Wmut (tracks 1, 2), isolated EN/D mutant (tracks 7, 8), isolated HspB7 (tracks 3, 4), and an equimolar mixture of AcdB7 and Wmut (tracks 5, 6) or EN/D mutant (tracks 9, 10) were run on SDS gel electrophoresis. Proteins were preincubated either in the absence (-) or in the presence (+) of GA. The positions of crosslinked complexes and molecular mass markers are indicated by arrows. Representative data of three independent experiments.

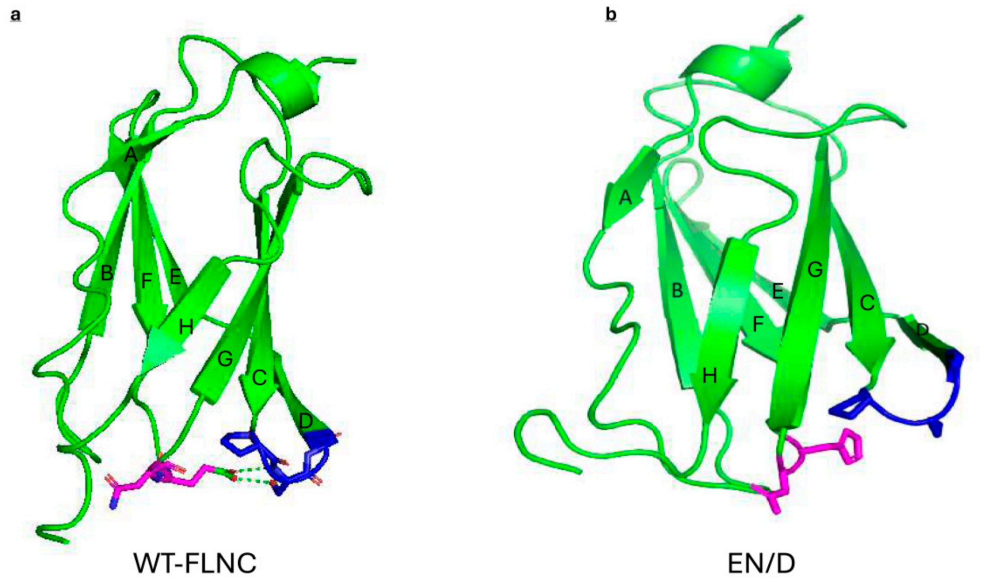

Figure S5. Alphafold 3 models of the 22nd immunoglobulin domain of the WT FLNC (a) (pTM 0.81) and its EN/D mutant (b) (pTm 0.81). In the case of the WT fragment, Glu 2472 forms hydrogen bonds (dashed lines) with Ser 2448 connecting two loops formed by strands C/D and F/G. The EN/D mutation prevents the formation of these hydrogen bonds.
